# The Axenic and Gnotobiotic Mosquito: Emerging Models for Microbiome Host Interactions

**DOI:** 10.3389/fmicb.2021.714222

**Published:** 2021-07-12

**Authors:** Blaire Steven, Josephine Hyde, Jacquelyn C. LaReau, Doug E. Brackney

**Affiliations:** ^1^Department of Environmental Sciences, Connecticut Agricultural Experiment Station, New Haven, CT, United States; ^2^Center for Vector Biology and Zoonotic Diseases, Connecticut Agricultural Experiment Station, New Haven, CT, United States

**Keywords:** axenic, gnotobiotic, mosquito, microbiome, model system

## Abstract

The increasing availability of modern research tools has enabled a revolution in studies of non-model organisms. Yet, one aspect that remains difficult or impossible to control in many model and most non-model organisms is the presence and composition of the host-associated microbiota or the microbiome. In this review, we explore the development of axenic (microbe-free) mosquito models and what these systems reveal about the role of the microbiome in mosquito biology. Additionally, the axenic host is a blank template on which a microbiome of known composition can be introduced, also known as a gnotobiotic organism. Finally, we identify a “most wanted” list of common mosquito microbiome members that show the greatest potential to influence host phenotypes. We propose that these are high-value targets to be employed in future gnotobiotic studies. The use of axenic and gnotobiotic organisms will transition the microbiome into another experimental variable that can be manipulated and controlled. Through these efforts, the mosquito will be a true model for examining host microbiome interactions.

## The Origins of an Axenic Mosquito Model

The term axenic refers to the growth of a single strain or species entirely free from contamination of any other organisms. The axenic state has also been referred to as “germ free.” We discourage this practice as it tends to implicate the microbes associated with a host with disease, ignoring the myriad of commensal and beneficial associations between microorganisms and their host. In its original usage, the term axenic was generally applied to cultures of bacteria or single-celled eukaryotes. As early as 1885, Louis Pasteur hypothesized the potential of an axenic animal host, although he was of the belief that the resulting axenic animal would not be viable (Pasteur, 1885). His views seemed to be borne out in 1896 when the production of axenic guinea pigs failed to survive past 13 days (Nuttall and Thierfelder, 1896). An axenic fly (*Calliphora vomitoria*) was first reported by Wollman in 1911, raising the potential for producing axenic insects (Wollman, 1911). With advancements in nutrition, handling facilities, and aseptic techniques, species, such as mice and fruit flies, are now routinely reared for multiple generations under axenic conditions, demonstrating that the axenic state is not a death sentence for the host organism (Sang and King, 1961; Smith et al., 2007; Douglas, 2018).

In 1930s, researchers turned their efforts to rear axenic mosquitoes, reporting the production of aseptic *Aedes aegypti* larvae in sterile media (Trager, 1935a,b, 1937). A series of studies would go on to refine the axenic techniques and were generally focused on determining the nutritional requirements of mosquito larvae (Lea et al., 1956; Singh and Brown, 1957; Akov, 1962; Lang et al., 1972; Rosales-Ronquillo et al., 1973). From the earliest studies, it was recognized that the microbiome was a potential source of essential nutrients. In 1935, Trager reported that sterile larval rearing media needed to be supplemented with particular nutrients, reporting that an essential ingredient in the media formulation was “heat-and alkali-stable…it seems to belong to the B group of vitamins” (Trager, 1935b). In fact, it was these studies that established a diet of liver and yeast extract for larval rearing, which is employed in many mosquito laboratories to this day. However, these studies occurred before the molecular revolution in microbiology in which we learned that the majority of microorganisms in the environment are often recalcitrant to laboratory cultivation (Hug, 2018; Lewis et al., 2021). Thus, there was some concern as to whether reports of axenic mosquitoes were reliable, as they may have been colonized by some of this biological “dark matter” (Rinke et al., 2013). Then, in 2014, a study called into question whether mosquitoes could be reared axenically at all, and proposed a potentially novel mechanism for the role of microbes in mosquito biology (Coon et al., 2014, 2017).

## Microbial Induced Hypoxia and Larval Development

It was recently reported that removing the mosquito larval microbiome by surface sterilizing eggs resulted in the production of axenic larvae that would expire in the first instar stage of development. However, larval development could be rescued if the larvae were provided with a live culture of a laboratory strain of *Escherichia coli* (Coon et al., 2014). In a follow-up experiment, the researchers performed a transposon mutant screen to identify the genetic determinants allowing for *E. coli* to rescue larval development. Through this screen, they identified that axenic larvae colonized by mutants in *cydB* and *cydD*, which encode cytochrome *bd* oxidase, failed to develop (Coon et al., 2017). The respiratory oxidase cytochrome *bd* allows *E. coli* to grow in oxygen-replete conditions, with maximal activity at oxygen concentrations of 25–50 nM (D’mello et al., 1996). This suggested an important role for this terminal electron transporter in bacteria-host interactions. Coon et al. observed that the guts of larvae colonized by wild-type *E. coli* showed a cyclical behavior in oxic conditions over development, with declining oxygen levels prior to molting. When colonized by the *cydB/cydD* mutants, gut anoxia did not manifest, and the larvae failed to develop. Furthermore, expression of mosquito-encoded hypoxia-inducible transcription factor was associated with larval development, supporting a connection between anoxia and larval growth (Valzania et al., 2018a). Therefore, the hypoxic model proposes that *E. coli* cytochrome *bd* mutants do not scavenge O_2_ from the gut lumen, thereby not producing anoxic conditions, and thus, the larvae failed to develop (Coon et al., 2017). This would require that microbes are not just a passive source of nutrients to the larvae, but a living respiring microbial population was necessary for larval development. These observations would make mosquitoes unique from other insects, like *Drosophila*, which are routinely reared in an axenic state and would seem to support Pasteur’s original thesis that some hosts may require a living microbiome for survival.

## Reemergence of Axenic Mosquito Models

In 2018, we reported the generation of axenic *Ae. aegypti* larvae that were capable of developing into adults. The sterility of the mosquito host was verified by both culture-dependent and culture-independent methods (Correa et al., 2018). More recently, Romoli et al. described a method to produce axenic *Ae. aegypti* larvae through a process of “transient colonization.” Briefly, axenic larvae are colonized by a genetically modified strain of *E. coli* that can be removed during larval development, referred to as decolonization. The decolonized larvae go on to produce axenic adult mosquitoes (Romoli et al., 2021).

**Figure 1:**
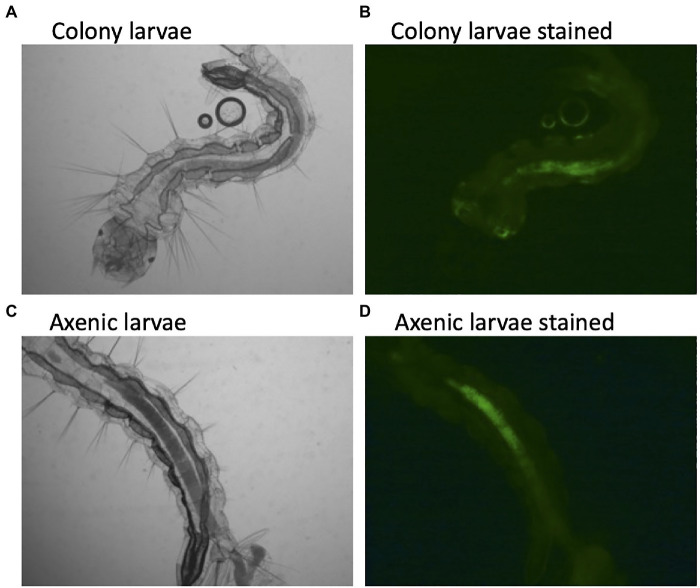
Hypoxic conditions in the guts of conventionally reared and axenic *Aedes aegypti* larvae. Larvae were reared to the third instar of development and stained with Image-iT Hypoxia Reagent, which fluoresces in anoxic conditions (<5% oxygen). Axenic larvae were reared in the dark to protect light sensitive B-vitamins in the culture medium. A subset of axenic larvae were subsequently verified to be free of microbial contamination by both culture-dependent and culture-independent (16S rRNA and fungal rRNA gene PCR) methods.

The description of axenic mosquitos seems to contradict the microbial-driven hypoxia model described above, which would require a living functional microbiome. Yet, several lines of evidence suggest that the hypoxic model may be incorrect. First, there was no difference in oxygen concentration between colonized larvae and decolonized axenic larvae (Romoli et al., 2021). Here, we show that axenic *Ae. aegypti* mosquitoes raised in the complete absence of a microbiome and stained with a fluorescent dye as a hypoxia marker maintain anoxic conditions in the gut (Figure 1). This indicates that mosquitoes themselves are capable of scavenging oxygen from the gut lumen. We thusly propose an alternative explanation to the observation that *E. coli* cytochrome *bd* mutants are unable to rescue larval development. Cytochrome *bd* mutants of *E. coli* demonstrate slower growth and produce lower biomass than their wild-type counterparts (Goojani et al., 2020). This suggests that the reproduction rate of cytochrome *bd* mutants may not be sufficient to support larval growth, not their ability to drawdown oxygen. In this view, the defects in the electron transport chain of *E. coli* lead to slower growth, which is then insufficient to support the high levels of metabolism required for larval development. A similar phenomenon has been observed in *Drosophila*, where it was reported that the quantity of bacteria present determined fly development and longevity rather than a particular microbial species or traits. In fact, the best predictor of how well a microbe would affect *Drosophila* development was how well that microbe grew on fruit fly culture medium (Keebaugh et al., 2018). This may also explain why *E. coli* K12, a laboratory-adapted bacterium, is still able to rescue larval development (Correa et al., 2018). *E. coli* K12 maintains high growth levels in larval growth media and thus can act as a source of larval nutrition, despite presumably losing many characteristics that would normally facilitate host colonization (Liu and Reeves, 1994; Browning et al., 2013).

Data from the newly developed axenic models also support a nutritional role of the microbiome. For instance, larvae reared on a high-density diet of liver and yeast extract have to be reared in the dark to protect a light-sensitive component in the food, presumably a B vitamin (Hyde et al., 2019a). Transcriptional data from decolonized axenic larvae showed that folate biosynthesis, folate transport, and thiamine metabolism were all upregulated in comparison with their microbially colonized cohorts, additionally pointing to a B vitamin deficiency in axenic larvae (Romoli et al., 2021). Furthermore, supplementing the diet of axenic larvae with folate increased larval survival 4-fold (Romoli et al., 2021). Finally, using data from the axenic and decolonized mosquito models, Wang et al. verified and replicated the observation that supplementing the diet of axenic larvae with B vitamins, in this case riboflavin, can rescue development. They further showed that axenic larvae undergo gut hypoxia when reared under appropriate conditions, verifying that microorganisms are not required to produce anoxic conditions (Wang et al., 2021a). Thus, axenic studies of mosquito larvae have come full circle, highlighting the role of the microbiome in supplying essential nutrients, B-vitamins, such as folate and riboflavin, as the key function of the microbiome in mosquito larval development.

## Are Microbes Essential for Larval Development?

The development of axenic larvae seems to suggest that living microorganisms are dispensable to larval growth. Yet, the microbes that make up the microbiome are clearly providing high amounts of biomass, food, and essential vitamins. It is unlikely any of these nutrients would be present in the environment in sufficient quantities to support larval growth in the absence of living microbes. In this regard, a living microbiome is likely required to support mosquito development in the wild. The fact that axenic larvae took longer to develop and produced smaller adults indicates that the axenic diet has not yet been optimized (Correa et al., 2018). In addition, several nutrients, such as amino acids and vitamin mixes, were lethal to the larvae when provided in high concentrations in the rearing water, which was only overcome when nutrients were provided in a semi-solid agar plug (Correa et al., 2018). This provides information on how mosquito larvae feed and acquire nutrients in the environment. Larval nutrition has long been recognized as a potential target for mosquito control and influences the development and fitness of adult mosquitoes (Souza et al., 2019; Dittmer and Gabrieli, 2020). Thus, the axenic model will continue to be important in defining and characterizing the nutritional requirements of mosquito larvae and the potential for identifying diet or microbial-based larval control programs.

## The Role of the Microbiome in Adult Mosquitoes

**Figure 2:**
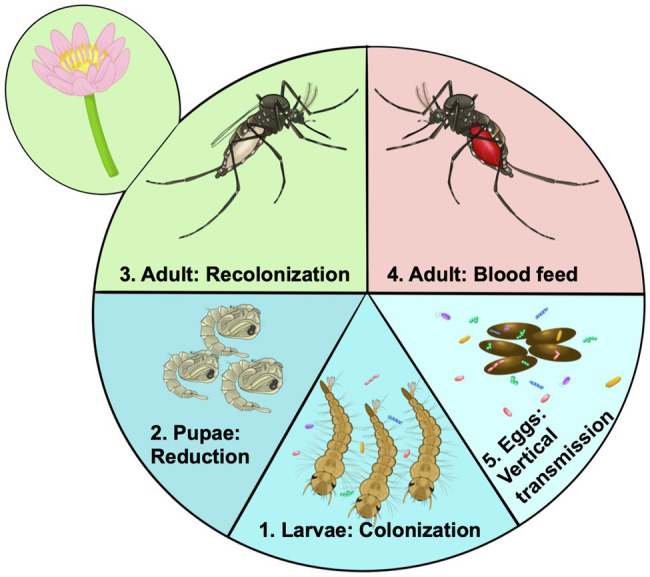
Acquisition and transmission of the microbiome over the life cycle of the mosquito. 1. Microorganisms from the aquatic environment colonize the larvae. These organisms provide essential nutrients such as B vitamins. 2. During pupation there is a large reduction for or even elimination of the microbiome. 3. After emergence mosquitoes are exposed to rearing water potentially being recolonized by aquatic bacteria, but are also exposed to new sources of colonizing bacteria such as flower nectar. 4. Certain members of the microbiome may facilitate blood digestion by female mosquitoes becoming more abundant in the gut. Other bacteria may be adapted to survive the high nutrients and reactive oxygen species. 5. Through colonizing the female ovaries microbes may be deposited on the egg surface, ensuring they are available to colonize newly hatched larvae, there by continuing the colonization cycle.

The microbiome of mosquito larvae is thought to be largely acquired from their aquatic environment (Coon et al., 2016b; Saab et al., 2020). As mosquitoes are holometabolous, going through a complete metamorphosis from larvae to adults through a “resting” pupal phase (Figure 2), larval nutrition and health have direct consequences on the fitness of the adult (Moller-Jacobs et al., 2014; Linenberg et al., 2016). Presumably, this means the status of the larval microbiome will also affect traits of the adult mosquito. Evidence suggests that many microbes are lost during metamorphosis when mosquitoes develop from larvae to pupae and emerge as adults (Lindh et al., 2008; Chavshin et al., 2015). The adult mosquito is exposed to new food sources, such as flower nectar, as well as being more mobile and able to visit other locations, and the anautogenous female mosquito requires a blood meal for reproduction (Figure 2). Thus, the microbiome of adult mosquitoes differs from larvae, in both composition and diversity (Muturi et al., 2017; Wang et al., 2018; Hyde et al., 2019b). Yet, no serious detrimental effects of the axenic state on adult *Ae. aegypti* mosquitoes have been documented. Axenic mosquitoes had similar, if not slightly longer lifespans than their bacterially colonized cohorts, and female mosquitoes took a blood meal, laid a similar number of eggs, and the eggs of the axenic *Ae. aegypti* females gave rise to viable offspring (Correa et al., 2018). Similarly, there were no detrimental effects of the axenic state on longevity of fecundity of decolonized mosquitoes (Romoli et al., 2021). Additionally, transcriptomic analysis of axenic adult mosquitoes showed a muted response in terms of gene expression change between axenic and conventionally reared mosquitoes, indicating similar physiological states for colonized and axenic mosquitoes (Hyde et al., 2020). Thus, it appears that the axenic state is not an obvious burden for the adult mosquito, at least in the case of *Ae. aegypti*.

Mosquitoes pose a significant and continued public health threat worldwide as they are responsible for transmitting numerous pathogenic viruses and parasites, such as dengue virus, West Nile virus, malaria parasites, and filarial nematodes, resulting in millions of infections each year. As such, there has been considerable research interest into the interactions between the mosquito microbiota and the pathogens they carry. Different microbial taxa have shown both positive and negative associations with the vectorial capacity of mosquitoes (Cirimotich et al., 2011; Guégan et al., 2018; Romoli and Gendrin, 2018; Caragata et al., 2019). Additionally, the composition and membership of the microbiome have been associated with a long list of other mosquito phenotypes. These include insecticide resistance, lifespan, and fecundity (Minard et al., 2013a; Dada et al., 2018; Wang et al., 2021b). Yet, all these studies have one thing in common, and they assume an interaction between microbiome members and host phenotypes. In this regard, these associations are not addressable with an axenic model, which can only investigate phenotypes in the presence or absence of a microbiome. What is required to link the status of the microbiome to host phenotypes is a model in which the diversity and composition of the microbiome can be mechanistically controlled and altered.

## A Defined Microbiome: Gnotobiotic Mosquitoes

The ability to perform the microbiome presence/absence studies is important, but they are binary in nature and, therefore, somewhat limited. The true power of axenic models is their ability to be manipulated so that defined microbiomes, whether it be an individual or multiple community members, can be introduced, thereby allowing the systematic examination of the effects that specific community members have on host biology. These systems, referred to as gnotobiotics, allow microbiome research to move beyond correlational studies to hypothesis-driven examinations of causation.

A gnotobiotic host is one in which every living organism in association with the host is defined. Consequently, gnotobiotic systems are limited by the availability of both an axenic host and microbiome community members that can be cultured. Despite these limitations, our ability to decipher the complexity of host-microbiome interactions has accelerated in recent years through advancements in genomics, proteomics, metabolomics, and culturing techniques. Initial studies in axenic flies revealed that developmental delays could be avoided through the introduction of a single community member and that certain community members were more effective at counteracting these delays (Bakula, 1969; Storelli et al., 2011). Functional characterization of these observations through a transposon mutagenesis screen and metagenome-wide association analysis determined that *Drosophila* larval development and adult fly metabolic homeostasis were significantly affected by the bacterial community members’ ability to synthesize pyrroloquinoline quinone, which is an important modulator of the flies insulin/insulin-like growth factor signaling (Shin et al., 2011; Chaston et al., 2014).

Monoculture gnotobiotics are a very useful tool for elucidating the contributions that the microbiota has on host development and physiology but are reductionist and cannot measure the influence of intra-community interactions on the system at large. Early on it was demonstrated that the microbiomes influence on host biology can be greater than the sum of its parts (Schaedler et al., 1965). Axenic flies colonized with communities of varying complexity reveal that some phenotypic traits are highly influenced by community interactions (Newell and Douglas, 2014; Gould et al., 2018). For instance, axenic *Drosophila* showed prolonged development times and elevated triglyceride contents in comparison with conventionally reared bacterial colonized flies. When the flies were recolonized with individual members cultivated from the microbiome, there were strain specific responses of the flies in triglyceride levels and longevity, but it was only when strains of both *Acetobacter* and *Lactobacillus* (the dominant members of the *Drosophila* microbiome) were presented to the axenic flies that the flies phenotypes returned to wildtype levels (Newell and Douglas, 2014).

Another approach for examining community scale effects and interactions is through transfer studies. While not truly gnotobiotic, transfer studies in which the microbiome of an individual with a specific disease state is introduced into an axenic host have become a useful tool for examining microbiome correlates of disease (Vrieze et al., 2012; Fei and Zhao, 2013; Schulz et al., 2014). For instance, the now famous study documents that transferring the microbiome from lean and obese mice results in increased capacity for energy harvest and weight gain for the mice receiving the “obese microbiome” (Turnbaugh et al., 2006). These systems have the benefit of more realistically capturing the complexity of interactions associated with the microbiome but suffer from an incomplete understanding of the members within the community and their individual contributions to host phenotypes.

Until recently, mosquito-microbiome studies have been limited due to the lack of an axenic model. Consequently, most studies have relied upon the use of antibiotics to clear the resident bacteria in order to interrogate microbiome-mosquito interactions. Using this approach, several groups have reported that the microbiome plays a role in modulating gut immunity thereby effecting susceptibility to viral and parasitic pathogens (Xi et al., 2008; Dong et al., 2009; Kalappa et al., 2018; Wu et al., 2019). Similarly, antibiotic clearance of the microbiota has demonstrated a role for the microbiome in mosquito metabolism and sensitivity to insecticides (Xiao et al., 2017; Barnard et al., 2019; Chabanol et al., 2020). These studies, like the presence/absence studies used in axenic models, offer generalizable insights into the effects of the microbiota on host phenotypes. While these studies support that the presence of a microbiome is linked to mosquito phenotypes, it is difficult to parse out microbiome impacts from those potentially associated with sustained antibiotic exposure. It has been demonstrated in mammalian systems that antibiotics can induce immunologic and metabolic changes in the host, inhibit eukaryotic translation, and alter mitochondrial function (Kalghatgi et al., 2013; Badal et al., 2015; Moullan et al., 2015; Yang et al., 2017; Gopinath et al., 2018). In addition to potential side effects, it has been shown that antibiotics do not eliminate resident microbiota, but rather cause a dysbiosis, as some members of the mosquito microbiome likely harbor antibiotic resistance (Hughes et al., 2014; Schubert et al., 2015; Hyde et al., 2019b). This may also explain contrasting results between studies that employ antibiotic clearance, as the net result may be an altered microbial composition rather than a comparison between the presence and absence of a microbiome. For instance, Xi et al. (2008) reported a significant reduction in dengue virus infection after antibiotic treatment of *Ae. aegypti* mosquitoes, whereas Audsley et al. reported no effect of antibiotic treatment on the permissiveness of *Ae. aegypti* to infection by dengue virus (Audsley et al., 2017). In addition, several studies have employed antibiotic clearance in attempts to recapitulate monoculture gnotobiotics in mosquitoes, by “clearing” the resident microbiota with antibiotics and then exposing the mosquitoes to a bacterium of interest. These studies demonstrate that specific bacterial community members may affect mosquito susceptibility to pathogens and host physiology (Dong et al., 2009; Apte-Deshpande et al., 2012; Ramirez et al., 2012; Hughes et al., 2014; Xiao et al., 2017; Wu et al., 2019). However, there is no way to separate the possible influence of antibiotics on these traits, or little to no verification that the mosquitoes are true gnotobiotics.

Recent efforts have been made to examine mosquito-microbiome interactions using axenic mosquitoes. Introduction of individual bacterial isolates at the larval stage revealed strain-specific effects on larval survivorship and development time as well as adult mosquito biometrics, such as body size and reproductive fitness as well as susceptibility to dengue virus and Zika virus (Coon et al., 2016a; Dickson et al., 2017; Correa et al., 2018; Carlson et al., 2020; Giraud et al., 2021). It has also been demonstrated that simplified communities can successfully colonize both axenic larvae and adult mosquitoes (Correa et al., 2018). Correa et al. found that the composition and function of the microbiome may be important determinants of phenotypic plasticity observed between individual mosquitoes. The generation of axenic/gnotobiotic mosquito models now make it possible to systematically interrogate the effects of the microbiome on mosquito biology without the use of antibiotics.

## Opportunities and Challenges for Axenic and Gnotobiotic Mosquitoes

The advent of the axenic/gnotobiotic mosquitoes opens up a wide range of questions that can be addressed by the research community. The method for generating and rearing axenic mosquitoes has been published and is achievable by any laboratory with the ability to maintain aseptic conditions, sterilize the required equipment, and perform basic microbiology (see Hyde et al., 2019a for a detailed protocol). To date, axenic rearing from larvae to adults has only been reported for *Ae. aegypti*, but gnotobiotic *Aedes atropalpus*, *Aedes albopictus*, *Anopheles gambiae*, *Culex quinquefasciatus*, and *Toxorhynchites amboinensis* have all been reported (Coon et al., 2016a,b, 2020; Valzania et al., 2018b). In this respect, gnotobiotic models may be achievable for a wider range of host species. Implementing these gnotobiotic studies will require an appropriate mosquito host and bacterial strains capable of supporting larval development.

Perhaps, one of the greatest opportunities for gnotobiotics is the potential for standardizing mosquito studies. It stands to reason that different research laboratories employing varied diets, rearing conditions, and being geographically separated are likely to harbor differing microbiome compositions in the mosquitoes they rear. Thus, at least a portion of variability reported between studies is likely due to heterogeneity in microbiome composition and structure between laboratories. Gnotobiotic models allow for standardization of the microbiome and transitioning the microbiome to a controlled variable. The creation of a defined tractable model microbiome, similar to the altered Schaedler flora employed in gnotobiotic mouse studies (Biggs et al., 2017), would be a resource that could be shared among researchers, and act as a baseline to investigate the consequences of microbiome manipulation on mosquito phenotypes. This will require identifying those bacteria that would serve the greatest utility for a defined microbiome.

## A Path Forward: A Most Wanted List for Microbes in Gnotobiotic Studies

**Figure 3:**
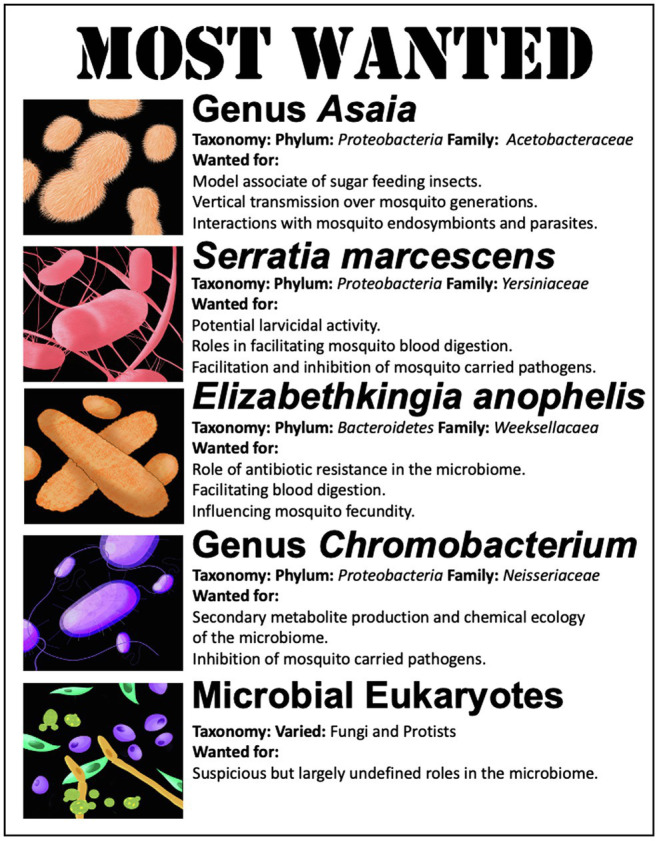
List of organisms of significant interest for gnotobiotic studies in mosquitoes.

In general, the diversity of the microbiome in an individual mosquito is rather low, being comprised of ~10–50 bacterial species (Minard et al., 2013a). Yet, there are certain microbial members that appear to be commonly associated with mosquitoes or that have been correlated to particular phenotypic outcomes, which makes them particularly attractive targets to investigate in a gnotobiotic model. Below, we list five microbiome members that are high-value targets for future gnotobiotic studies (Figure 3). This is by no means an extensive list of microbiome members that may play a role in mosquito physiology but is a review of some of the mosquito microbiome members that show the greatest promise for untangling host microbe interactions or potential microbes that could be employed in microbial-based mosquito borne disease control. Notably, not included on the list is the bacterial endosymbiont *Wolbachia*. This maternally inherited organism may be the most well-studied mosquito-associated bacterium, with over 100 years of active study (Kaur et al., 2021). However, the difficulty in growth and maintenance of *Wolbachia* in pure culture, its biology, host range, and inheritance makes it a difficult organism to employ in gnotobiotic studies (Voronin et al., 2010; Hughes et al., 2012). Despite the myriad of effects *Wolbachia* plays in mosquito biology, reproduction, and control of mosquito carried pathogens (Turley et al., 2009; Hancock et al., 2011; Iturbe-Ormaetxe et al., 2011; Jiggins, 2017), it does not make the list.

### Genus *Asaia*

Bacteria in the genera *Asaia* (phylum Proteobacteria) are acetic acid bacteria within the family Acetobacteraceae. Acetic acid bacteria are differentiated from other bacteria as they are obligate aerobes that oxidize sugars, sugar alcohols, and ethanol with the production of acetic acid as the major end product (Raspor and Goranovič, 2008). Bacteria of the genus *Asaia* colonize multiple insects, across multiple orders, such as the Diptera, Hymenoptera, Hemiptera, and Homoptera (Crotti et al., 2010). This includes mosquitoes within *Aedes* sp., *Anopheles* sp., and *Culex* sp. (Crotti et al., 2009; Freece et al., 2014; Ramos-Nino et al., 2020). *Asaia* is among the numerically dominant bacterial populations that colonize mosquitoes as assessed by sequencing surveys (Chouaia et al., 2010; Damiani et al., 2010) and can be found in larval and adult mosquitoes and multiple tissues (e.g., the adult gut, testes, ovaries, and salivary glands; Favia 2007, 2008; Mancini, 2018). Geographically dispersed, *Asaia* has been identified in mosquitoes from Brazil (Oliveira et al., 2020), Canada (Novakova et al., 2017), Iran (Rami et al., 2018), Italy (Alfano et al., 2019), Kenya (Osei-Poku et al., 2012), Madagascar (Minard et al., 2013b), and the United States (Muturi et al., 2017), among others. Species of *Asaia* are often found in flower nectar, the food source of newly emerged mosquitoes (Bassene et al., 2020). They can also spread through mosquito populations by paternal transmission during mating (Damiani et al., 2008). *Asaia* may use their colonization of the female reproductive tract to ensure vertical transmission through a process of egg smearing, whereby they colonize the egg surface in order to be ingested by newly hatched larvae (Damiani et al., 2010). The importance of the bacteria to the larvae is demonstrated by the fact that reduction of *Asaia* bacterial load by treatment with antibiotics slows larval growth. Subsequently, supplementing larval diets with *Asaia* bacteria accelerated larval development time (Mitraka et al., 2013). This suggests that these bacteria are able to meet the nutritional needs of developing larvae (Chouaia et al., 2012). Evolutionary analysis of *Asaia* genomes indicates that these organisms have underwent a process of genome reduction as they became associated with insects, yet preserved an insecticide degrading gene, pyrethroid hydrolase (Comandatore et al., 2021). Thus, beyond providing a source of nutrition to developing larvae, *Asaia* may play a role in protecting mosquitoes from insecticides.

*Asaia* bacteria appear to interact with the endosymbiotic bacteria and parasites carried by mosquitoes. When the mosquito microbiome was supplemented with *Asaia* bacteria, there was an observed impediment to the vertical transmission of *Wolbachia*, a mosquito endosymbiont, suggesting inter-species competition (Hughes et al., 2014). Additionally, several studies have documented a negative relationship between *Asaia* bacteria and *Plasmodium*, the causative agent of malaria (Capone et al., 2013; Cappelli et al., 2019a). It is thought that *Asaia* bacteria prime an immune response that prevents the malarial parasite from developing in the mosquito (Cappelli et al., 2019a). In this regard, the *Asaia*-mosquito symbiosis may be a relationship with significant public health implications.

These data and observations show that *Asaia* sp. colonize multiple mosquito hosts as well as other sugar-feeding insects. *Asaia* sp. are present across multiple life stages, in multiple tissues, and are reliably inherited between mosquito generations. *Asaia* bacteria are also amenable to genetic modification and mosquito recolonization, which makes them an attractive tool for studying the genetic determinants of mosquito colonization (Favia et al., 2007). Additionally, because bacteria in the genus *Asaia* are generally non-pathogenic they are attractive targets for paratransgenic strategies for mosquito vector control. Thus, *Asaia* is on the list of most wanted as they represent an ideal model bacterium to study an apparent beneficial relationship between a bacterium and the mosquito host.

### Serratia marcescens

*Serratia* (phylum Proteobacteria) is a genus of facultatively anaerobic bacteria within the family Yersiniaceae. Bacteria in the genus *Serratia* are common among the bacteria that make up the mosquito microbiome (Sharma et al., 2020). Several different species of *Serratia* have been identified in mosquitoes, including *Serratia odorifera* (Apte-Deshpande et al., 2012), *Serratia nematodiphila* (Patil et al., 2012), and *Serratia fonticola* (Chen and Walker, 2020, 14). Yet, one bacterium in particular has received a considerable research focus as a mosquito associate, *Serratia marcescens*.

*Serratia marcescens* is a cosmopolitan bacterium with multiple environmental reservoirs, including soil and water (Abreo and Altier, 2019). It is often found in hospital settings and is a significant cause of nosocomial infections (Mahlen, 2011; Khanna et al., 2013). Mosquitoes are also an environmental reservoir. Strains of *S. marcescens* have been found to colonize mosquito larvae, the adult midgut, female ovaries, and male accessory glands, and on the surface of newly laid eggs (Tchioffo et al., 2016; Wang et al., 2017). Various isolates of *Serratia* demonstrate larvicidal activity. A strain of *S. nematodiphila* demonstrated high mortality to several mosquitoes species: *C. quinquefasciatus* (100%), *Anopheles stephensi* (95%), and *Ae. aegypti* (91%) after 48 h of exposure (Patil et al., 2012). Similarly, gnotobiotic *Ae. aegypti* mosquito larvae colonized with a strain of *S. marcescens* experienced >85% mortality, and the surviving larvae took approximately twice as long to develop (Correa et al., 2018). The characteristic red coloration of *S. marcescens* may play a role in its antagonism to larval development. Prodigiosin, the red pigment produced by *S. marcescens* shows larvicidal activity when introduced to larvae (Patil et al., 2011; Suryawanshi et al., 2015).

In contrast, *S. marcescens* seems to play a beneficial or at least neutral role in the adult mosquito. Gnotobiotic adult *Ae. aegypti* mosquitoes colonized by *S. marcescens* showed no increase in mortality (Correa et al., 2018). Similarly, no fitness defects were noted for adult *An. gambiae* or *Culex pipiens* mosquitoes colonized by *S. marcescens* (Koosha et al., 2019; Ezemuoka et al., 2020). In fact, *S. marcescens* may participate in mosquito blood digestion. The genome of *S. marcescens* encodes several genes for heme uptake and storage, as well as demonstrating alpha-hemolytic activity, i.e., the complete lysis of blood cells (Chen et al., 2017). Yet, fewer females infected with *S. marcescens* took blood meals in comparison with their uninfected cohorts (Kozlova et al., 2021).

*Serratia marcescens* has also been shown to interact with the capacity of mosquitoes to transmit disease. For instance by inhibiting *Plasmodium* development in the mosquito, thereby reducing the spread of malaria (Seitz et al., 1987; Bahia et al., 2014; Bai et al., 2019). Yet, there is high intra-specific diversity between *S. marcescens* strains capable of inhibiting *Plasmodium*. This indicates the anti-parasitic ability is likely due to a small number of genetic determinants, such as flagellum biosynthesis, that may not be conserved among all members of the species (Bando et al., 2013). Genome sequencing of mosquito-associated strains of *S. marcescens* showed various virulence factors and antibiotic production which may be involved in controlling pathogen infection of the mosquito (Chen et al., 2017). A recently described class of natural products, the stephensiolides, was isolated from a mosquito-associated *Serratia.* It is posited that these compounds have antimicrobial properties, which may also antagonize *Plasmodium*, as well as facilitate motility and transfer of bacterial cells within and between mosquitoes (Ganley et al., 2018). Additionally, colonization of the mosquito with *S. marcescens* induces gene expression changes in pathways, such as peptidoglycan recognition receptors that may enhance anti-parasitic immune responses that act to inhibit *Plasmodium* (Stathopoulos et al., 2014). In contrast, mosquito colonization by *S. marcescens* has been reported to increase the infection rate of mosquitoes by particular arboviruses. Specifically, *S. marcescens* colonization has been linked to increases in the infection of mosquitoes with dengue-2 and chikungunya virus (Apte-Deshpande et al., 2012). This increased permissiveness to viral infection is thought to occur through bacterial excretion of a protein *Sm*Enhancin, which interferes with mucins of the mosquito gut epithelia, allowing dissemination of viral particles (Wu et al., 2019). In this regard, *S. marcescens* appears to inhibit or facilitate the vector competence of mosquitoes depending on the pathogen under consideration.

*Serratia marcescens* is on the target of most wanted mosquito which associates to study in gnotobiotic systems due to its complex interactions with the mosquito host. Colonization outcomes vary widely between larvae and adults. The potential relationships and mechanisms driving interactions between *S. marcescens* and the pathogens vectored by mosquitoes vary from the general, such as a priming of the immune system to the specific production of metabolites that aid or hinder pathogen infection or spread. In this respect, there is a rich list of potential host microbe interactions and phenotypic outcomes to characterize in regard to the association between *S. marcescens* and mosquitoes.

### Elizabethkingia anophelis

*Elizabethkingia* (phylum Bacteroidetes) is a genus within the family Weeksellaceae. This bacterium is unique on this list as its origins are from mosquitoes, being first isolated from the midguts of *An. gambiae* (Kämpfer et al., 2011). However, the genus *Elizabethkingia* is considered to be ubiquitous in the environment. Although genomic-based analyses suggest that the mosquito-associated strains form a distinct evolutionary sublineage within the *Elizabethkingia anophelis* species complex (Breurec et al., 2016).

*Elizabethkingia anophelis* is an opportunistic human pathogen, causing pneumonia, bacteremia, neonatal meningitis, nosocomial bacteremia, and neutropenic fever (Lau et al., 2016). Cases of bacteremia are often associated with poor clinical outcomes, with mortality rates as high as 23.5% (Lau et al., 2016). Cases of *E. anophelis* infections have been reported from Singapore, Hong Kong, Taiwan, and the United States (Janda and Lopez, 2017). Because of the association between *E. anophelis* and mosquitoes, it was initially posited that mosquitoes may be a vector (Frank et al., 2013). Subsequent studies suggest that *E. anophelis* cases are far more likely to be hospital acquired infections, although sporadic, community-acquired cases have been reported (Hayek et al., 2013; Perrin et al., 2017; Lee et al., 2021). One characteristic that makes *E. anophelis* infections particularly challenging in the clinical environment is that the bacterium is resistant to multiple antibiotics including cephalosporins, aminoglycosides, and carbapenems (González and Vila, 2012; Breurec et al., 2016). A notable 112 predicted proteins identified in the genome of mosquito-associated strains of *E. anophelis* were annotated to features involved in resistance to antibiotics or other toxic compounds (Kukutla et al., 2013). Indeed, in recent surveys of antibiotic-resistant bacteria in the mosquito microbiome, isolates of *Elizabethkingia* were identified to possess multi-drug resistance against ampicillin, carbenicillin, gentamycin, tetracycline, and kanamycin (Hyde et al., 2019b; Ganley, 2020). It is not clear what, if any, role this multi-drug resistance may play in colonizing the mosquito host, other than a potential fitness advantage against other bacteria making up the mosquito microbiome.

*Elizabethkingia anophelis* shows differential preferences for mosquito hosts. The bacterium showed high colonization rates for *An. gambiae* and *A. stephensi* but was rarely detected in *Aedes triseriatus* (Chen et al., 2015). Like other bacteria on this list, *Elizabethkingia* sp. can be found in high numbers in the mosquito ovaries and may be vertically transmitted from mother to offspring (Akhouayri et al., 2013). Yet, colonization by *Elizabethkingia* sp. may induce melanotic lesions in the fat bodies of mosquito larvae and adults, suggesting a potential antagonistic relationship (Akhouayri et al., 2013). *E. anophelis* may be particularly important in mosquito blood digestion. Cell counts of *E. anophelis* increased approximately 3-fold in the guts of post-blood fed mosquitoes, and animal erythrocytes promoted *E. anophelis* growth in cell culture (Chen et al., 2015, 2020). The bacterium also displays hemolytic activity and encodes several hemolysins that may participate in the digestion of erythrocytes in the mosquito gut, along with antioxidant genes, which could provide defense against the oxidative stress that is associated with blood digestion (Kukutla et al., 2014). Mosquitoes colonized with *E. anophelis* produced more eggs than did those treated with erythromycin or with a standard microbiome, suggesting *E. anophelis* may increase mosquito fecundity, potentially by increasing available nutrients from the blood meal (Chen et al., 2020).

*Elizabethkingia anophelis* makes this list as it is a true mosquito associate that may shed light on specific genomic adaptations for colonizing the mosquito host. Furthermore, with the high levels of antibiotic resistance, *E. anophelis* is a model to study competition among microbiome members. Finally, *E. anophelis* appears to play a significant role in blood digestion a key point in the mosquito lifecycle.

### Genus *Chromobacterium*

The genus *Chromobacterium* (phylum Proteobacteria) is facultatively anaerobic bacteria withing the family Neisseriaceae. Like the other bacteria on this list, species of *Chromobacterium* are abundant in the environment and can be readily isolated from soils and water sources (Batista and da Silva Neto, 2017). Much of the research interest in C*hromobacterium* has been driven by the biotechnological and pharmaceutical importance of secondary metabolites produced by the strains, which include antibiotics, quorum sensing molecules, lipopolysaccharides, and the pigment violacein that gives *Chromobacterium violaceum* its characteristic purple coloration (McClean et al., 1997; Durán and Menck, 2008; Kothari et al., 2017).

*Chromobacterium* strains are not particularly abundant in the mosquito microbiome, although they are among bacterial members commonly identified in several species of mosquito (Minard et al., 2013a). Instead, the research focuses on the *Chromobacterium* predominately derives for their role in control of mosquito populations and their interactions with mosquito vector competence. Various strains have shown detrimental effects of mosquito survival, lifespan, blood feeding, and fecundity (Gnambani et al., 2020). Colonization of *Ae. aegypti* or *An. gambiae* with a strain of *Chromobacterium* isolated from mosquito midguts resulted in rapid mortality of both larvae and adults (Ramirez et al., 2014). Larvae exposed to sublethal doses of the bacterium had lengthened developed time, suggesting chronic effects of even small populations of the bacteria (Short et al., 2018a). In the adult mosquito, *C. violaceum* exposure decreased the proportion of females seeking a blood meal, significantly reduced the numbers of eggs laid, and reduced hatches from the resulting eggs (Gnambani et al., 2020). A preparation of a strain of *Chromobacterium* with no living cells maintained strong lethal effects in mosquitoes, indicating this bacterium was producing one or several bioactive compounds (Caragata et al., 2020). Transcriptional analysis of mosquitoes with chromobacterium exposure revealed gene expression changes in pathways related to detoxification, xenobiotic response, and stress response, similar to that of an insecticide exposure (Short et al., 2018b). The same strain was also observed to produce hydrogen cyanide in larval water at sufficient concentrations to induce larval mortality, offering another possible mechanism for larvicidal activity (Short et al., 2018a). The effects of chromobacteria exposure can be transgenerational with the offspring of exposed females showing developmental delays and increased mortality (Short et al., 2018b). The genome of *Chromobacterium vaccinii*, another bacterium with potential roles in mosquito biocontrol, encodes several genes for virulence factors that may explain their toxicity, and these include siderophores, production of hydrogen cyanide, as well as multiple chitinase genes (Vöing et al., 2020).

*Chromobacterium* sp. are among the microbes that show inhibitory activity against the pathogens carried by mosquitoes. Chromobacteria cell extracts and cultures show anti-pathogen activity outside of the mosquito host, indicating the potential production of secreted metabolites inhibiting pathogen growth (Ramirez et al., 2014). For example, violacein, the violet pigment compound produced by many species of *Chromobacteria*, is potent antimicrobial with antiparasitic activities against *Plasmodium* (Lopes et al., 2009). A specific compound produced by *Chromobacterium* sp. Panama, romidepsin a histone deacetylase inhibitor, also showed high activity against *Plasmodium* (Saraiva et al., 2018b). Additionally, the *Chromobacterium* sp. Panama produces a protease that attacks the envelope protein of dengue virus, thereby blocking its ability to bind to and infect cells (Saraiva et al., 2018a). Thus, these observations point to the wealth of chemical compounds produced by species of C*hromobacterium* and their potential to influence the biology, behavior, and vector competence of their mosquito hosts.

The genus *Chromobacterium* makes the list based on the assortment of potentially bioactive compounds that are produced by these bacteria. In this regard, these bacteria offer a unique insight into the chemical ecology of the mosquito microbiome.

### Microbial Eukaryotes

The microbiome of mosquitoes consists of more than just bacteria. Yet, there is a significant knowledge gap concerning the single-celled eukaryotes that inhabit the mosquito microbiome.

Fungal diseases are common in insects, including mosquitoes. These entomopathogenic fungi have been extensively described elsewhere (e.g., Scholte et al., 2004; Kanzok and Jacobs-Lorena, 2006; Shen et al., 2020). However, it is not so clear that these organisms can be considered part of the normal microflora of the mosquito microbiome. Far less is known of the commensal fungi that are common residents of the microbiome and are the next set of organisms on the most wanted list. A survey of culturable fungal isolates among laboratory reared and field caught mosquitoes found fungal isolates in the class *Microbotryomycetes* to be common among field caught mosquitoes, but absent in the microbiome of laboratory-reared mosquitoes (Hyde et al., 2019b). This suggests fungi may play an important role in the microbiome, but normal colony conditions may not be favorable to commensal fungi (Hyde et al., 2019b). As to the role fungi may play in the environment, certain fungi may exert their effect on the microbiome by attracting gravid females to a breeding site. Particularly, yeasts produce CO_2_ and other volatile compounds through fermentation, which can signal to the mosquito, a suitable habitat with sufficient sugar and microbial resources to support larval development (Malassigné et al., 2020). In this manner, these fungi can ensure colonization of the newly hatched larvae (Reeves, 2004). Gnotobiotic larvae colonized by *Saccharomyces cerevisiae* develop normally, indicating yeast can supply all the required nutrients for larval development (Correa et al., 2018). Certain fungi, such as *Cladosporium*, *Aspergillus*, *Ampullimonas*, and *Cyberlindnera*, actively participate in digesting the fructose that mosquitoes ingest from flower nectar (Guégan et al., 2020). Thus, fungi appear to play roles in both larval and adult nutrition. Other yeasts, such as *Wickerhamomyces anomalus*, colonize the reproductive organs of both male and female mosquitoes, indicating the potential for vertical transmission between generations, suggesting a stable multi-generational association (Ricci et al., 2011). Several fungi have also shown potential in inhibiting the pathogens carried by mosquitoes. For instance, mosquitoes colonized by microsporidian fungi demonstrated a significant decline in *Plasmodium* development in the mosquito, potentially through priming the mosquito immune response to the malarial parasite (Bargielowski and Koella, 2009; Herren et al., 2020). Similarly, protein toxins produced by the yeast *Wickerhamomyces anomalus* inhibit *Plasmodium* and other entomopathogenic fungi (Cappelli et al., 2019b). In contrast, fungi of the species *Talaromyces* may promote mosquito infection by dengue virus through suppression of the digestive enzyme trypsin (Angleró-Rodríguez et al., 2017). In this regard, fungi clearly have the potential to play a multitude of roles in the mosquito microbiome, yet remain an enigma.

Beyond the protist parasite *Plasmodium*, there is very little knowledge concerning whether mosquitoes harbor a stable population of protists in their microbiome (Guégan et al., 2018). Belda et al. employed a method based on peptide-nucleic acid clamps to suppress amplification of host DNA and specifically interrogate the eukaryotic members of the mosquito microbiome (Belda et al., 2017; Taerum et al., 2020). The eukaryotic microbiome of larval samples was dominated by the *Ichthyosporea* group, a lineage of unicellular organisms that includes parasites and commensals of a wide range of animals (Glockling et al., 2013). Similarly, metabarcoding and sequencing of the 18S rRNA genes from mosquitoes in Thailand identified *Ascogregarina* as the dominant microbial eukaryote in the mosquito microbiome (Thongsripong et al., 2018). This protist has been shown to have a range of fitness consequences on host mosquitoes ranging from detrimental to neutral (Erthal et al., 2012). Yet, the organism displays the hallmarks of a parasitic infection as oocysts are ingested from the larval water, enter epithelial cells, and use host cell mitochondria to supply the energy required to mature (Chen and Wu, 1997). *Trypanosoma brucei*, a protist parasite and causative agent of trypanosomiases, normally carried by tsetse flies, can survive in mosquito midguts for up to 48 h. Co-infection of mosquitoes with *Trypanosoma* and *Plasmodium* increased the malarial parasite load in the mosquito, potentially increasing the risk of malarial spread (Dieme et al., 2020). Thus, there is evidence that the microbiome of the mosquito may host a population of protists, but their roles and interactions with the host are essentially undescribed.

The microbial eukaryotes carried by mosquitoes represent a virtually uncharted territory for discovery in the mosquito microbiome. As such, they are the final members to make the list of high-value targets for gnotobiotic studies.

## Community Ecology and Microbiome Interactions

Bacteria are unlikely to find themselves in the mosquito as a monoculture. Instead, mosquitoes are colonized by a community of interacting individuals and populations.

Microbial interactions may be parasitic, where one organism benefits at the cost of another: mutualistic, such that both organisms benefit, or commensal, when one organism benefits at no cost or benefit to the other. These interactions are facilitated by mechanisms, such as metabolite exchange, cross-feeding, and antibiotic production (Phelan et al., 2012; Pacheco and Segrè, 2019). Thus, the properties of the community are determined by the separate functional contributions from each species and their interactions. In this respect, the attributes of a community are difficult to predict from the traits of its members when they are reared in a mono-culture (Sanchez-Gorostiaga et al., 2019). It is increasingly apparent that the microbiome of mosquitoes acts as a community, rather than a collection of individuals. Co-occurrence networks based on bacterial census data identified multiple pairwise and higher order interactions, indicating an interwoven and linked microbial community (Hegde et al., 2018). More direct evidence of microbial interactions within the mosquito has also been documented. For example, when a strain of *S. marcescens* was introduced to *Ae. aegypti* larvae as a monoculture, 89% of the larvae died. The same strain inoculated in a simple three-member community reduced mortality to 50%. Yet, *Serratia* was identified among all of the assayed mosquitoes, suggesting that the larvicidal activity of *Serratia* is attenuated by the presence of other microbes (Correa et al., 2018). Microbial interactions also influence the digestion of mosquito food sources. The fructose that mosquitoes obtain through feeding on flower nectar can be digested in a trophic interaction involving both fungi and bacteria (Guégan et al., 2020). In another example, it was shown that when *E. anophelis* was co-cultured with a strain of *Pseudomonas* in the midguts of mosquitoes, *E. anophelis* upregulated gene products for heme degradation. This activity presumably facilitates blood digestion in the mosquito but also produced a metabolite of the class biliverdin, which may inhibit *Pseudomonas* growth. In this manner, *E. anophelis* gains a competitive advantage and may indirectly benefit the mosquito host (Ganley, 2020). These observations all point to the importance of viewing the mosquito microbiome as a community and taking a population ecology viewpoint when linking the status of the mosquito microbiome to host phenotypes. Thus, an important step going forward will be to employ gnotobiotic mosquitoes as a resource to characterize microbiome interaction networks.

## Conclusion and Perspectives

The development of axenic and gnotobiotic mosquito models offers the potential to transform the study of the mosquito microbiome. These models transition microbiome studies from correlational associations between microbes and their host to controlled experiments that can systematically manipulate the composition, genetics, and biochemistry of the of the microbiome. In this manner, the mechanistic underpinnings of the relationship between the mosquito and its microflora can begin to be uncovered. The wealth of studies that have already linked the microbiome to mosquito biology and the diseases they carry have already provided an abundance of hypotheses to test and will be an excellent foundation for future studies. We have provided a list of potential microbiome members that represent particularly high-value targets for future gnotobiotic studies, but they are only the forefront of a broad field of investigation. Recently, researchers proposed the formation of a “Mosquito Microbiome Research Consortium” and laid out recommendations for best practices for collecting, analyzing, and sharing mosquito microbiome data (Dada et al., 2021). We propose that properly designed and controlled axenic and gnotobiotic studies should be central pillars to a unified effort to disentangle the role of the microbiome in mosquito biology and microbe-mosquito control programs.

## Author Contributions

BS and DB contributed to manuscript conception and writing. JH contributed to data for Figure 1 and manuscript editing. JL contributed to figure production. All authors contributed to the article and approved the submitted version.

### Conflict of Interest

The authors declare that the research was conducted in the absence of any commercial or financial relationships that could be construed as a potential conflict of interest.
